# Numerical and Functional Responses of Forest Bats to a Major Insect Pest in Pine Plantations

**DOI:** 10.1371/journal.pone.0109488

**Published:** 2014-10-06

**Authors:** Yohan Charbonnier, Luc Barbaro, Amandine Theillout, Hervé Jactel

**Affiliations:** 1 INRA, UMR 1202 BIOGECO, Cestas, France; 2 Univ. Bordeaux, BIOGECO, UMR 1202, Pessac, France; 3 LPO Aquitaine, 433 chemin de Leysotte, Villenave d'Ornon, France; INRA-UPMC, France

## Abstract

Global change is expected to modify the frequency and magnitude of defoliating insect outbreaks in forest ecosystems. Bats are increasingly acknowledged as effective biocontrol agents for pest insect populations. However, a better understanding is required of whether and how bat communities contribute to the resilience of forests to man- and climate-driven biotic disturbances. We studied the responses of forest insectivorous bats to a major pine defoliator, the pine processionary moth *pityocampa*, which is currently expanding its range in response to global warming. We used pheromone traps and ultrasound bat recorders to estimate the abundance and activity of moths and predatory bats along the edge of infested pine stands. We used synthetic pheromone to evaluate the effects of experimentally increased moth availability on bat foraging activity. We also evaluated the top-down regulation of moth population by estimating *T. pityocampa* larval colonies abundance on the same edges the following winter. We observed a close spatio-temporal matching between emergent moths and foraging bats, with bat activity significantly increasing with moth abundance. The foraging activity of some bat species was significantly higher near pheromone lures, i.e. in areas of expected increased prey availability. Furthermore moth reproductive success significantly decreased with increasing bat activity during the flight period of adult moths. These findings suggest that bats, at least in condition of low prey density, exhibit numerical and functional responses to a specific and abundant prey, which may ultimately result in an effective top-down regulation of the population of the prey. These observations are consistent with bats being useful agents for the biocontrol of insect pest populations in plantation forests.

## Introduction

Predator-prey relationships are shaped by the functional and numerical responses of the predators to prey density [1]. The numerical response involves predator density being adjusted to prey abundance through changes in reproduction, dispersal and foraging behaviour leading to aggregative patterns in habitat patches with large feeding resources [2]. The functional response is the adjustment of the predator consumption rate to the abundance or the biomass of its feed so that the consumption rate is a major determinant of the top-down regulation of prey population by predators [3], [4]. Insect pests are a major threat to forests worldwide and it is expected that climate change will further enhance insect herbivory, due to positive response of forest insects to warmer and drier conditions [5], [6]. Climate warming is also likely to trigger both more intense and severe insect outbreaks, and range expansion northwards and upwards [7]–[9]. The pine processionary moth *Thaumetopoea pityocampa* Denis & Schiffermüller (Lepidoptera: Notodontidae) is the main defoliator of pines (*Pinus* spp) in the western part of the Mediterranean Basin, including Southern Europe, the Balkans and North Africa. Defoliation by this species significantly reduces tree growth, and severe or repeated defoliation can lead to tree death [10]. The distribution range of *T. pityocampa* is currently expanding towards higher latitudes and elevations as a result of the release of thermal constraints allowing improved winter survival and feeding activity [5], [11].

Predators and parasitoids contribute to shaping the cyclic population dynamics of *T. pityocampa* [12], [13]. However, recent studies have also suggested that climate warming leads to more stable top down regulation involving generalist predators rather than specialist predators [1], [14], [15]. Birds have long been considered as the only predatory vertebrates that are efficient pest regulators, although recent comparative studies in tropical forests have shown that insect predation by bats may be more significant than predation by birds [16]–[18]. Using mostly echolocation, insectivorous bats are nocturnal predators preying on invertebrates both at forest edges and within forest stands. Using diverse hunting techniques they can catch a wide range of arthropod prey and consume over half of their body mass in insects nightly [19]. Given that many herbivorous insects are mainly active at night [20], it is therefore likely that chiropterans contribute greatly to pest regulation [21]. However, the pest regulation service provided by bats remains to be quantified, notably for the control of those forest pests that represent a growing concern under climate change [18]. A pioneer review on the biological control of forest insects by vertebrates pointed out the lack of information on numerical response of predators to prey density, a key issue to better estimate predation effectiveness [22]. Since this seminal work, few if any studies on vertebrate predators have managed to fully address these critical questions [21]. Even if they revealed a significant impact of bat predation on insect population, all previous studies have failed to identify which prey insect species was actually concerned [21].

Species-specific prey consumption and foraging observations have been performed to study bats as insect predators but these two approaches have never been used simultaneously. Few studies to date have actually considered bat activity as a numerical response to insect prey availability [23]–[25]. Even fewer studies have investigated the functional responses of bats to insect abundance via dietary and faeces analyses [26], [27]. Here, in an innovative experimental approach, we used synthetic sex pheromone lures to manipulate the local availability of a specific prey species, the pine processionary moth (*Thaumetopoea pityocampa*), without modifying local environmental conditions, by contrast with the use of exclosures [21] or light attraction [28]. This method allowed investigating, in natural field conditions, the responses of bats to experimentally controlled prey availability. In this manipulative experiment, we formulated the following three hypotheses for bat predation on *T. pityocampa*: (i) the occurrence and abundance of predatory bats will match in time and space with those of the main insect prey species; (ii) predatory bats will increase their foraging activities when and where prey is more abundant, i.e. exhibiting positive numerical and functional responses to prey density; and (iii) insect prey abundance will decrease at the next generation where bat foraging activity has been higher, due to consumption of adult moths and consequent reduction of reproductive success.

## Materials and Methods

### Study area and site selection

The study area was located in the Landes de Gascogne forest (N44 45.000, W0 50.00), in south-western France; this forest is the largest forest plantation in Europe with approximately one million ha of pure stands of the maritime pine *Pinus pinaster*. The climate is thermo-Atlantic and the soil is podzolic. We selected 23 pine plantations of the same age (30 years old) with similar edges, at least 200 m long, and separated by at least 900 m. Edges in front of clearcuts or very young stands were avoided. The Office National des Forêts and Groupama provided us special authorizations to access their private stands and conduct the study.

### Moth surveys

**Figure 1:**
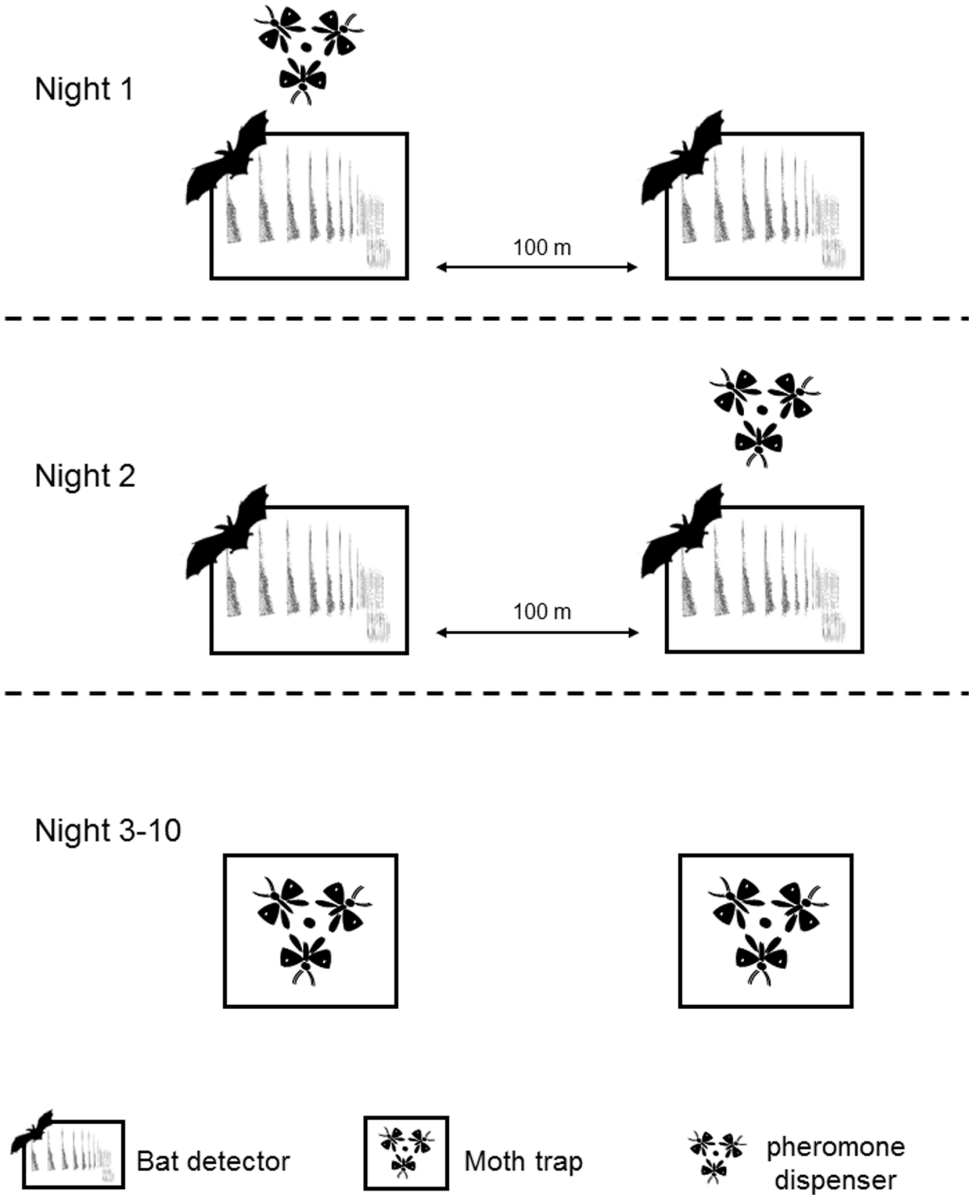
Experimental set up established in a subset of 12 pine plantation edges.

The field experiment was conducted in July 2012 during the period of peak *T. pityocampa* moth emergence [29]. Synthetic sex pheromone trapping is considered to be an efficient method to monitor *T. pityocampa* populations [30]. It relies on a positive relationship between pheromone trap captures and local population density estimated through counts of larval colonies [30]. After two nights of bat sampling (Figure 1), we replaced bat detectors with pheromone-baited traps at the same sites along the selected forest edges. These traps were 30×30 cm, plate sticky traps with both sides covered with glue, baited with 0.5 mg of pityolure [30]. Traps were activated for six consecutive nights. At the end of the trapping session, the sum of male captures was recorded for each trap to estimate the abundance of *T. pityocampa* in each plot.

In late February 2013, we estimated the density of *T. pityocampa* larval colonies along the same sampled stand edges in order to quantify the effect of adult moth predation by bats on the abundance of prey offspring. We counted all larval colonies on all pine trees along 100 m of stand edge, focusing on the first two rows of the stand where most of *T. pityocampa* larval colonies are concentrated [31]. We calculated the ratio of the number of larval colonies per stand edge divided by the number of male catches on the same edge as an indicator of moth reproductive success (because the sex-ratio is 1∶1 in *T. pityocampa*, the number of male captures approximated the number of pairs). Two stands were discarded because they had been thinned in between male trapping and larval colonies counting (for a total of N = 21 stands). Time patterns of *T. pityocampa* moth activity per night period were adapted from the detailed biological study of Demolin (1969).

### Bat surveys

Along the edge of each stand, one automatic ultrasound bat detector system (Sound Meter SM2BAT, Wildlife Acoustics) fitted with multidirectional microphones (SMX-US weatherproof ultrasonic microphone, Wildlife Acoustics) was installed to record night bat activity. Detectors were calibrated to detect all bat calls and programmed to record from one hour before sunset to one hour after sunrise. Each edge was sampled for two consecutive nights between July 9 and July 27, 2012. Recordings were included in the analysis only for nights without rain, when the wind was <30 km/h and the ambient temperature above 10°C [32]. In addition, we studied a subset of 12 forest edges where two bat detectors, separated by 100 m, were deployed: each of the two detectors was equipped with a *T. pityocampa* sex pheromone dispenser on alternate nights (Figure 1). The synthetic pheromone was used to attract and increase availability of flying male moths around the microphone of the bat detector.

Bat calls were identified by one trained operator (YC) using Batsound 4.1. We used existing identification keys and published data [33]–[35]. All sequences were analyzed and identified to the finest taxonomic level possible: species level identification was feasible for various kinds of calls, but there were large overlaps between some species making species level identification impossible. In the study area *Pipistrellus kuhlii* and *P. nathusii* calls were really similar. However, in our case, they were only allocated to *Pipistrellus kuhlii* because this species is very common and widespread within the study area, whereas *P. nathusii* is very rare according to regional atlas data [36] and it was never detected by diagnostic calls in our own survey. By contrast, using a conservative approach, we decided not to discriminate *Eptesicus serotinus* and *Nyctalus leisleri* and classified them as a single sonotype [37]. Both species were recorded with certainty (5012 calls for *E. serotinus* and 459 calls for *N. leisleri*) and both species are commonly distributed in the study area. Nevertheless it seems that serotine bats *E. serotinus* are far more abundant than lesser noctules *N. leisleri* in pine plantation forests at the regional level [36].

We assessed bat activity levels using the number of search phase sequences for all species or sonotype. These sequences were composed of two or more pulse calls separated from other calls by one second or more [37], [38]. To better evaluate bat activity, continuous sequences longer than 5 s were scored as two sequences. We also assessed bat feeding activity by counting the number of feeding buzz calls indicating active prey capture attempts by a foraging individual bat. Compared to regular calls, feeding buzz calls were defined as more steeply frequency-modulated with pulse intervals gradually decreasing [39].

### Statistical analyses

We used Generalized Linear Mixed Models (GLMM) [40] implemented in R-package lme4 [41] to analyze the complete data set of 23 edges during all sampled nights and thereby assess the effects of *T. pityocampa* abundance on bat activity. The response variable was the number of call sequences during each sampled night. The explanatory variable was *T. pityocampa* abundance. We accounted for the hierarchical structure of the data by adding nested random effects of night, plot and hour to the model intercept. Residual repartition and deviation from normality (normal Q–Q) interpretation plots were used for model validation. We also tested the individual effect of the presence of a *T. pityocampa* pheromone dispenser on bat activity, prey capture attempts and bat species richness. We paired echolocation data recorded with and without a pheromone lure, during the same night along the same edge in 12 forest stands, using a permutation t-test implemented in the R-package Deducer [42]. We log-transformed the ratio of larval colonies/male moth captures to meet the assumption that residuals are normally distributed. Thereby we used linear models to test for the effect of overall bat activity or bat feeding activity on moth reproductive success.

## Results

### Temporal patterns of bat and moth activity

**Figure 2:**
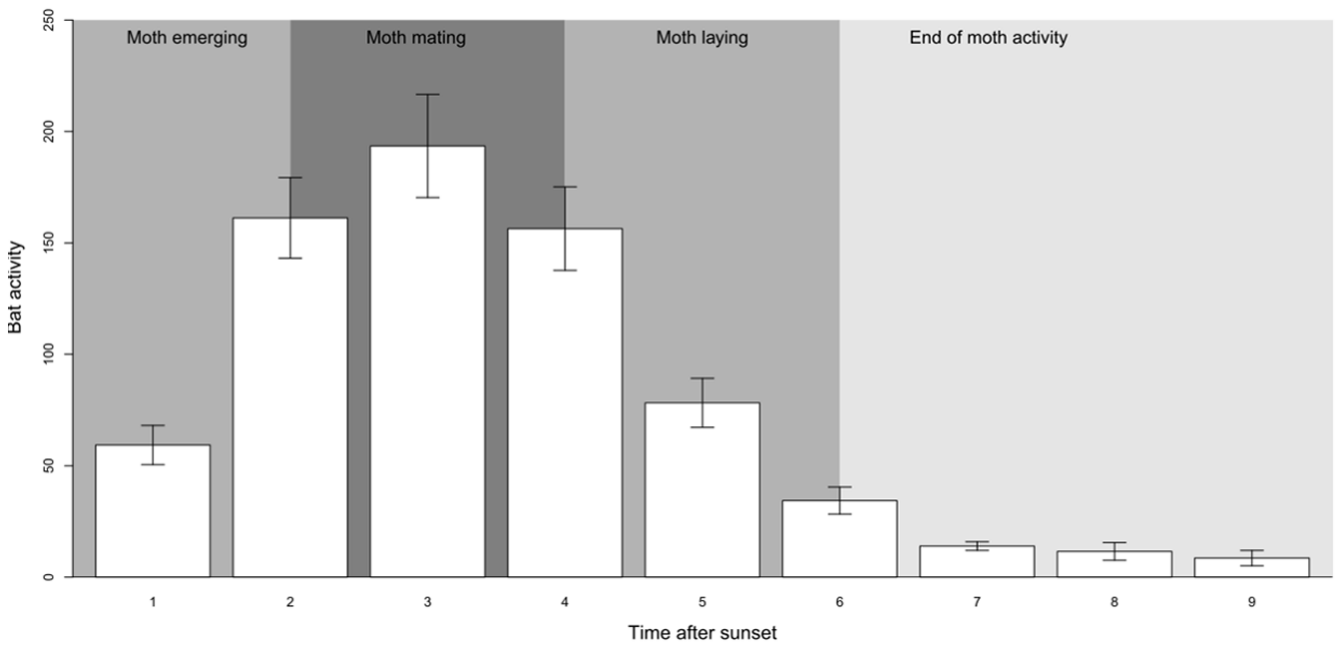
Compared periods of nocturnal activities for bats and pine processionary moths (adapted from Demolin 1969) along the 23 sampled forest edges.

We recorded 49271 passes identifiable to species or sonotype level. They include 27997 (56.9% of the identifiable passes) *P. kuhlii* passes, 17300 (35.1%) *E. serotinus - N. leisleri sonotype* passes, and 3015 (6.1%) *P. pipistrellus* passes. The other 1.8% were assigned to *Myotis* spp (341 passes), *Plecotus* spp (299), *Nyctalus noctula* (109), *Barbastella barbastellus* (94), *Nyctalus lasiopterus* (13) and *Rhinolophus ferrumequinum* (1). The maximum activity of bats along the 23 sampled edges matched the period of pine processionary moth mating activities, which occur during the 4 hours after sunset (Figure 2). Total bat activity and total feeding buzzes per night were significantly correlated (R^2^ = 0.47; *P*<0.0001) and these two variables showed the same temporal patterns throughout the night. Finally, bat activity rhythm, all species together, matched the described time pattern activity of *T. pityocampa*.

### Bat response to moth abundance

We caught 409 male moths in the 46 traps (mean 9 moths per trap). The mean capture per trap and per plot ranged from 1 to 22 suggesting that our experimental design fitted a range of *T. pityocampa* densities. For bat data analysis, we only used data from the first 4 hours of the night, i.e. when bat activity was maximal, in order to limit bias and to avoid zero-inflated counts. Total bat activity significantly increased with increasing moth abundance on forest edge (z = 17.27; *P*<0.0001) (Table 1). The activity of several bat species also significantly increased with local moth abundance: *P. kuhlii* (z = 18.41; *P*<0.0001), *E. serotinus - N. leisleri* sonotype (z = 4.154; *P*<0.0001) and *P. pipistrellus* (z = 7.684; *P*<0.0001) (Table 1). There was no effect of moth abundance on bat species diversity (z = 1.438; *P* = 0.151).

**Table 1:** Results of Poisson GLMMs linking total bat activity and individual species activities to pine processionary moth abundance.

Bat species	Estimates	SE	*z*-value	*P*
Total bat activity	0.0611	0.0035	17.27	<0.0001
*Pipistrellus kuhlii*	0.0973	0.0052	18.41	<0.0001
*Eptesicus serotinus - Nyctalus leisleri*	0.0215	0.0051	4.15	<0.0001
*Pipistrellus pipistrellus*	0.0909	0.0118	7.68	<0.0001
*Plecotus* spp	−0.0580	0.0426	−1.36	0.1732
*Barbastella barbastellus*	0.0127	0.0691	0.18	0.8550

### Bat response to moth aggregates

**Figure 3:**
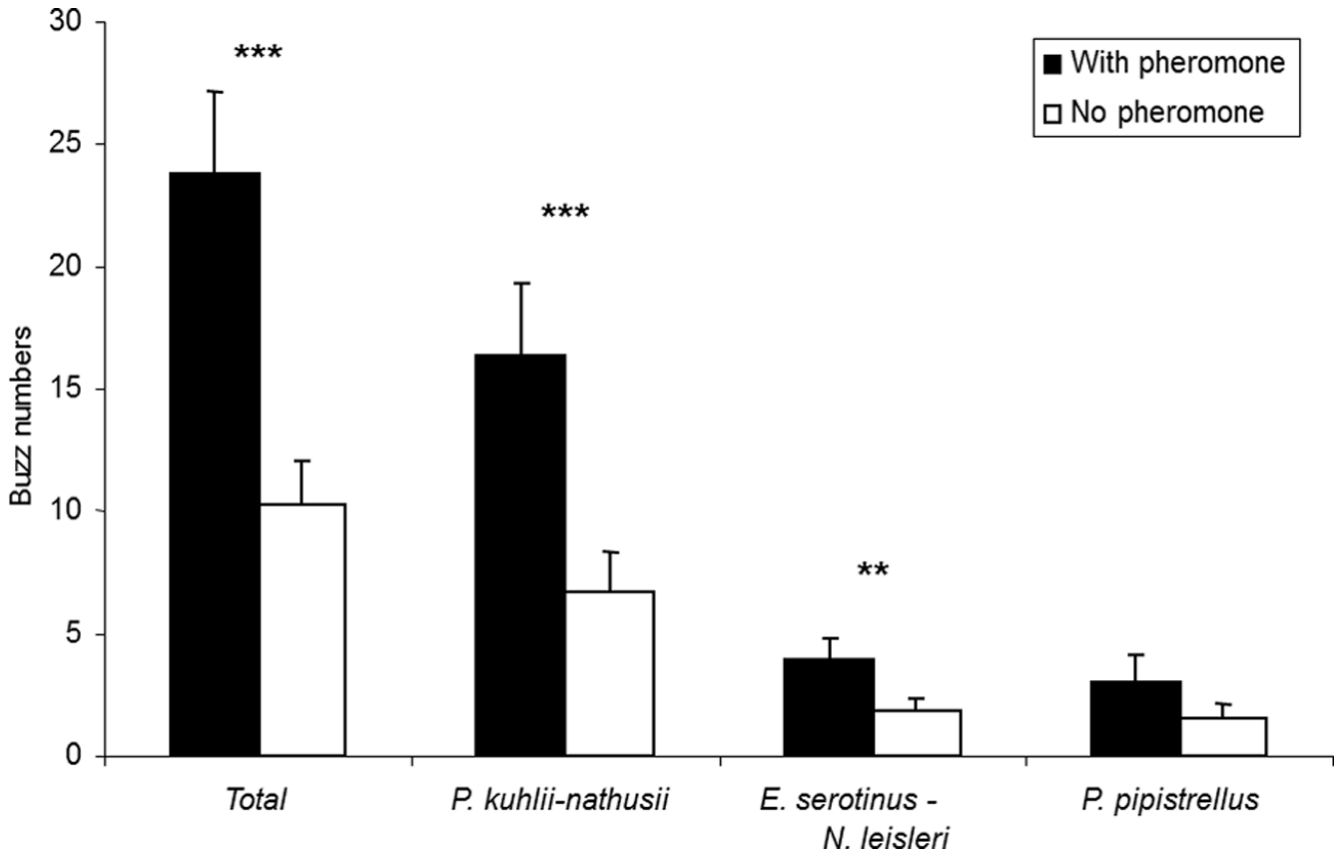
Comparison of prey capture attempts (mean number of buzzes+SE) for total and bat species groups in the presence (black bars) vs. in absence (white bars) of a *T. pityocampa* sex pheromone lure.

For a given edge within a given night, we did not observe any significant effect of the presence of pheromone lure on total bat activity (*P* = 0.558), or on the activities of *P. kuhlii* (*P* = 0.871), *E. serotinus - N. leisleri* sonotype (*P* = 0.422) and *P. pipistrellus* (*P* = 0.510). Artificial moth aggregates, resulting from pheromone attraction, had also no significant effect on bat species richness (*P* = 0.856). However, even if bat activities remained the same along the edge, there was a significant increase in bat feeding activity near the pheromone lure, i.e. close to artificial moth aggregates (*P*<0.001; mean of differences = −13.58, see Figure 3). The largest bat species emitted significantly more feeding buzzes in the presence of pheromone lure (*E. serotinus - N. leisleri* sonotype; *P* = 0.013; mean of differences = −2.08; and *P. kuhlii; P* = 0.001; mean of differences = −9.62), while it was not the case for the smallest species *P. pipistrellus* (*P* = 0.214).

### Bat predation effect on moth demography

**Figure 4:**
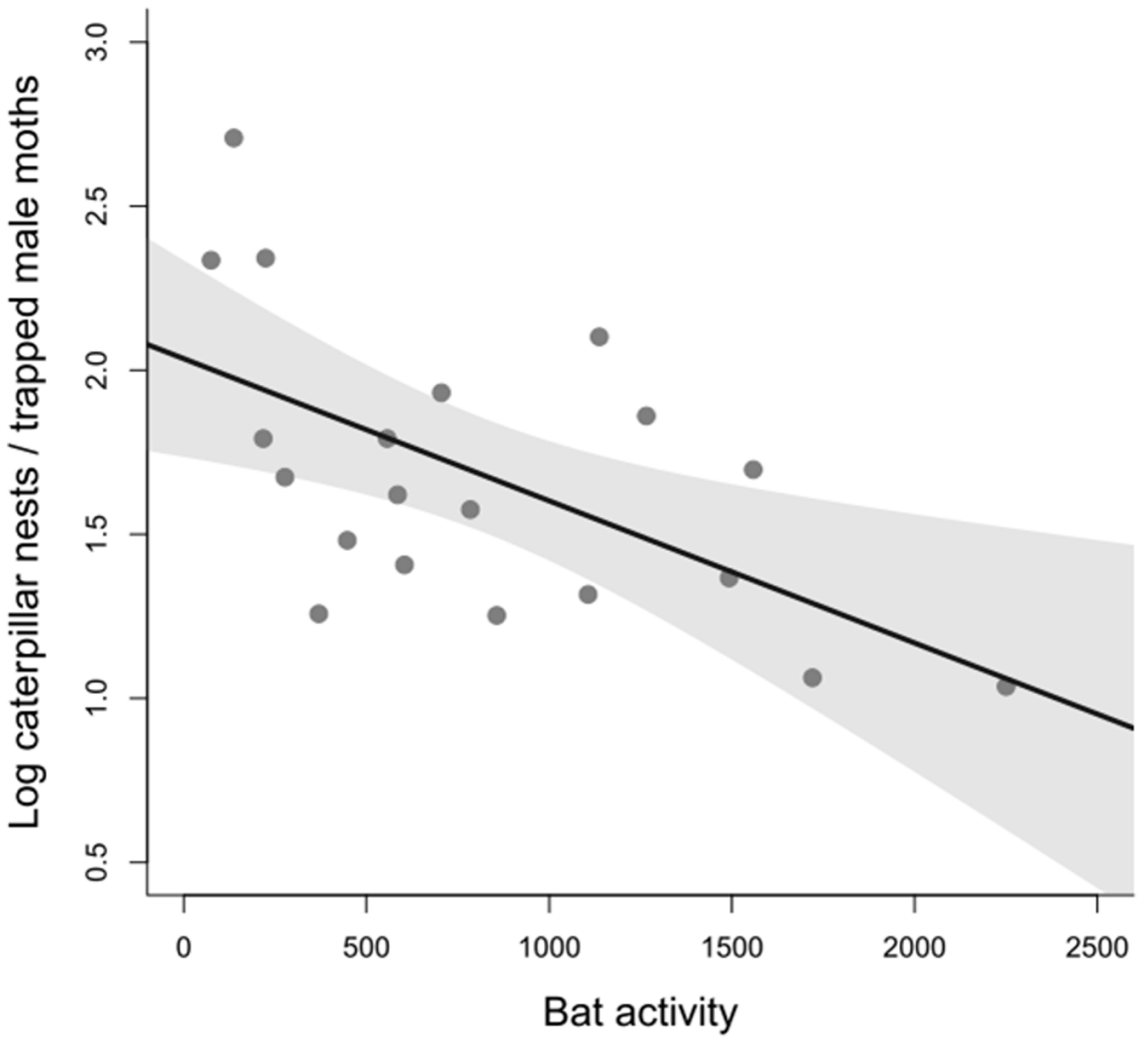
Effect of total bat activity on prey reproductive success (ratio of larval colonies per male moth captured) the following summer along 21 sampled edges.

There was a positive correlation between male moth abundance as estimated by pheromone trap catches and larval colonies abundance the next year along the same forest edges (R^2^ = 0.245; P = 0.013). More interestingly, the ratio of larval colonies per trapped males, a measure of fertility, significantly decreased with increasing total bat activity (R^2^ = 0.335; P = 0.003, see Figure 4) and the activity of the *P. kuhlii* (R^2^ = 0.166; *P* = 0.047) and the *E. serotinus - N. leisleri* sonotype (R^2^ = 0.418; *P*<0.001). By contrast, the activity of *P. pipistrellus* had no significant effect on moth abundance (R^2^ = 0.014; P = 0.270). Bat feeding activity did not show a significant effect on prey reproductive success (R^2^ = 0.102; P = 0.087). The ratio of larval colonies per trapped males significantly decreased with increasing *E. serotinus - N. leisleri* sonotype feeding activity (R^2^ = 0.391; P = 0.001).

## Discussion

The magnitude of arthropod consumption by bats changes along their reproductive cycle. In Europe, most of the bat species give birth in late spring or early summer [43]. In common with other mammals, lactation required a substantial energy expenditure [27], [44]. Female bats therefore have to optimize their hunting activities when lactating. In the study area, bats lactate in late June and July, and this is also the period when the emergence of *T. pityocampa* peaks. In early summer, *T. pityocampa* is the most abundant moth species in pine plantation forests (Charbonnier unpub. data), moreover showing a circadian rhythm in tight coincidence with bat activity (Figure 2). Furthermore, this temporal synchronization may be coupled with spatial matching due to the forest edge preference of both bats [23], [45], [46] and *T. pityocampa* adult moths [47]. Previous studies have shown that insectivorous bat activities are strongly correlated with arthropod abundance, suggesting that bats actively search for areas of concentrated prey resources [23], [25], [48]. In our study, we found that bat activity increased with prey availability (moth abundance, Table 1). This finding provides support for our initial hypothesis of significant bat numerical responses to *T. pityocampa* abundance. Our results are consistent with previous studies showing that insectivorous bats are able to adjust their predatory activity to prey availability [26], [27]. This behaviour may favour optimal foraging on resources that are unpredictable in time and space, such as local outbreaks of *T. pityocampa* within large pine plantations. Moreover, as the period of *T. pityocampa* emergence coincides with the period of highest energy requirements for female bats, it is possible that bats have also a demographic response to the moth. Further experiments are needed to test this hypothesis, as observed for another predatory vertebrate, the Eurasian hoopoe *Upupa epops* [49].

McCracken et al. [27], by including comparisons of bats’ diet vs. potential prey abundance in a large study area, suggested that there were bat functional responses to particular types of prey at the landscape scale. Here, without changing environmental conditions, we artificially increased the availability of one prey species in its favoured habitat using specific sexual pheromones. These lures may have several effects besides local attraction of males by increasing either abundance or flight activity in intensity and time. Introducing synthetic lures can shift or extend the flight period of male moths, so that increased bat capture attempts could result from the longer time window when bats are able to find the moths. This increase of prey availability, due to an increasing encounter probability, actually resulted in a significantly higher bat feeding activity (Fig. 2). This suggests that the more *T. pityocampa* moths are present on a given edge, the more foraging bats are able to feed on them. Without changing their overall flight activity at the local scale of a given forest edge, but only by enhancing prey capture attempts in relation to prey availability, bats seemed to display a functional response to this specific insect prey. We were unable to determine the exact shape of the functional response curve, because we used a proxy of feeding activity (buzz numbers) rather than true prey capture attempts per individual bat. Previous studies showed that Holling type II (cyrtoid) responses are the most frequent for vertebrates [50]. Nevertheless, bats can travel long distances, use different foraging tactics and feed on several prey, so it is more likely that the bat functional response is a Holling type III (sigmoid) response [51]. This type of functional response is mostly associated with generalist predators because they are able to switch between alternative prey items according to availability. Therefore, these bat populations may remain fairly abundant when *T. pityocampa* availability declines, and respond rapidly when the pine defoliator build up its populations.

Only the largest bat species showed a significant functional response. The *E. serotinus - N. leisleri* and *P. kuhlii* displayed significantly more prey capture attempts in the presence of higher moth availability. By contrast, the smaller *P. pipistrellus* did not produce more buzzes in the presence of a pheromone dispenser and thus moth availability. This species may be too small to feed on *T. pityocampa* and, according to the optimal foraging theory, would spend too much time handling this kind of prey [52]. Various other bat species specialize on moths, such as *Barbastella barbastellus* [53], [54] or *Plecotus* spp [55], but they did not show either numerical or functional responses to higher densities of moths in our study. These species can fly slowly and accurately to glean foliage-resting moths in dense vegetation [56] within old pine and mixed forests [57]; this may explain their lack of response to the experimentally generated flying moth along forest edges. Another explanation could be the low level of *T. pityocampa* populations during the experiment. Generalist species are known to have greater impact on prey populations at low density whereas specialized predators are more effective with high prey density [58], [59]. Here, moth density may have been too low to trigger a functional response by a bat specialist. In addition, if fruit bats commonly use olfactory cues to find their food, such behaviour is much less frequent in insectivorous bats, which use odour for social interactions rather than for foraging [60], [61]. It is therefore unlikely that the higher bat feeding activity recorded near sex pheromone lures was a direct response to prey odour (kairomonal attraction). Bats are polyphagous predators and there is no evidence that any are sufficiently specialized to have evolved the capacity to smell insect pheromones.

Jactel et *al.* [30] found a positive relationship between the abundance of *T. pityocampa* male moths trapped and the numbers of larval colonies in the next generation. In our study, we observed a significant and negative correlation between general bat activity during the flight period of male processionary moths (time period of the experiment) and the ratio of larval colonies (the next winter) per male moth trapped along the same forest edge. This finding clearly suggests that bat numerical and functional responses to moth density resulted in less offspring per adult moth. It is therefore most likely that bats were feeding on *T. pityocampa* moths, reducing the reproductive success of the insect species. This prediction is strengthened by low predation and parasitism rates of egg masses in the study area [62], which is the only intermediate life stage between laying moths and larval colonies. Nevertheless, these results are preliminary and would require a longer period of monitoring to be generalized. Several records of bats caught in pine processionary pheromone traps (Martin, unpublished data) and visual observation of bats feeding (e.g. [63]; Serra-Cobo, unpublished data) also suggest that several bat species can actively prey on *T. pityocampa* moths. However only an in depth study of bats diet in pine forests, for example based on DNA analysis of droppings, will allow to formally confirm this assumption. Moreover, monitoring the effects of bat predation on moth demography during an entire epidemic cycle would allow testing the hypothesis that top down regulation is less effective during pest outbreaks [58], [59]. Such an impact of predation on adult moths is critical for the effectiveness of top down regulation of the pest insect because this stage of the biological cycle is the smallest in size [64]. In *T. pityocampa*, many other biological control agents including predatory vertebrates (birds) have been described for all larval and pupal development stages, but few before for adult moths [12], [13].

It is increasingly acknowledged that bats may contribute substantially to pest regulation in temperate agro-ecosystems [21]. However, the reasons for this effectiveness are not fully understood. Insectivorous bats are an example of generalist predators that maintain constant vital rates and stable populations by shifting to alternative prey [1], [14]. Even if our study was limited to a single year, our new experimental approach based on artificial increase in prey availability using pheromone lures revealed a facilitating mechanism of foraging plasticity: the ability of bats to detect and concentrate on local aggregates of the most abundant prey species. These numerical and functional responses of generalist bat species may result in a negative effect on the reproductive success of the prey and then in a reduction of the population growth rate, at least in condition of low prey density. These various characteristics make bats potential biological control agents that could contribute to regulate *T. pityocampa* populations.
