# Correction: Numerical and Functional Responses of Forest Bats to a Major Insect Pest in Pine Plantations

**DOI:** 10.1371/journal.pone.0117652

**Published:** 2015-01-27

**Authors:** The PLOS ONE Staff

There is an error in the fourth sentence of the Abstract. The genus of the scientific name of the moth was omitted. The journal apologizes for this error that occurred while the manuscript was being prepared for publication. The sentence should read: We studied the responses of forest insectivorous bats to a major pine defoliator, the pine processionary moth *Thaumetopoea pityocampa*, which is currently expanding its range in response to global warming.
